# Similarities in cardiometabolic risk factors among random male-female pairs: a large observational study in Japan

**DOI:** 10.1186/s12889-022-14348-6

**Published:** 2022-10-28

**Authors:** Naoki Nakaya, Kumi Nakaya, Naho Tsuchiya, Toshimasa Sone, Mana Kogure, Rieko Hatanaka, Ikumi kanno, Hirohito Metoki, Taku Obara, Mami Ishikuro, Atsushi Hozawa, Shinichi Kuriyama

**Affiliations:** 1 Tohoku Medical Megabank Organization, Tohoku University, 2-1 Seiryo-machi, 980-8573 Sendai City, Japan; 2 Tohoku University Graduate School of Medicine, 2-1 Seiryo-machi, 980- 8575 Sendai City, Japan; 3 Department of Occupational Therapy, School of Health Sciences, Fukushima Medical University, 1 Hikariga-oka, 960-1295 Fukushima City, Japan; 4 Division of Public Health, Hygiene and Epidemiology, Tohoku Medical and Pharmaceutical University, 4-4-1 Komatsushima, 981-8558 Sendai City, Japan; 5 Department of Pharmaceutical Sciences, Tohoku University Hospital, 1-1 Seiryo- machi, 980-8574 Sendai City, Japan; 6 Department of Disaster Public Health, International Research Institute of Disaster Science, Tohoku University, 2-1 Seiryo-machi, 980-8573 Sendai City, Japan

**Keywords:** Anthropometric traits, Cardiometabolic risk factors, Diseases, Lifestyle habits, Random male-female pairs

## Abstract

**Background:**

Previous observational studies have shown similarities in cardiometabolic risk factors between spouses. It is still possible that this result reflects the age similarity of spouses rather than environmental factors of spouses (e.g. cohabitation effect). To clarify the importance of mate cardiometabolic risk factors for similarity of environmental factors, it is necessary to examine whether they are observed in random male-female pairs while maintaining the age of the spousal pairs. This study aimed to determine whether the similarities found between spousal pairs for cardiometabolic risks were also observed between random male-female pairs.

**Methods:**

This cross-sectional study included 5,391 spouse pairs from Japan; data were obtained from a large biobank study. For pairings, women of the same age were randomly shuffled to create new male-female pairs of the same age as that of the original spouse pairs. Similarities in cardiometabolic risk factors between the random male-female pairs were analysed using Pearson’s correlation or age-adjusted logistic regression analyses.

**Results:**

The mean ages of the men and women were 63.2 and 60.4 years, respectively. Almost all cardiometabolic risk factors similarities were not noted in cardiometabolic risk factors, including the continuous risk factors (anthropometric traits, blood pressure, glycated haemoglobin level, and lipid traits); lifestyle habits (smoking, drinking, and physical activity); or diseases (hypertension, type 2 diabetes mellitus, and metabolic syndrome) between the random male-female pairs. The age-adjusted correlation coefficients ranged from − 0.007 for body mass index to 0.071 for total cholesterol. The age-adjusted odds ratio (95% confidence interval) for current drinkers was 0.94 (0.81 − 1.09); hypertension, 1.07 (0.93 − 1.23); and type 2 diabetes mellitus, 1.08 (0.77 − 1.50).

**Conclusion:**

In this study, few similarities in cardiometabolic risk factors were noted among the random male-female pairs. As spouse pairs may share environmental factors, intervention strategies targeting lifestyle habits and preventing lifestyle-related diseases may be effective.

## Background

According to the American Heart Association’s Task Force on Risk Reduction, traditional risk factors for cardiovascular diseases include the following cardiometabolic risk factors: obesity and high blood pressure, high cholesterol levels, smoking, impaired glucose tolerance, left ventricular hypertrophy, and low levels of high-density lipoprotein cholesterol [1]. Searching for modifiable factors among these cardiometabolic risk factors and their modifications may lead to the primary prevention of cardiometabolic diseases. Furthermore, in recent years, the association of genetic factors with cardiovascular disease risk has been found [2–6].

Previous observational studies have shown similarities in cardiometabolic risk factors between spouses such as blood pressure (BP) [7–13], cholesterol levels [8–10, 12, 13], triglycerides levels [8, 10, 12], abnormal glucose tolerance [7, 8, 10–15], and smoking [9, 14]. Spousal concordance may be mainly explained by assortative mating and cohabitation effects [16]. Assortative mating is the tendency of people to select mates who bear greater similarities in characteristics such as discernible traits and behaviours (phenotypic assortment) or social and environmental factors (social homogamy). This causes an initial similarity between spouses. Cohabitation effects could be due to common environmental factors shared by couples or due to “partner interaction effects,” with partners influencing each other’s behaviour [17–19]. If concordance is mainly due to a cohabitation effect, then it might increase with the increase in the partnership duration [20]. Assortative mating and/or cohabitation effects may indicate a higher degree of similarities between a spouse’s lifestyle and the associated phenotyping (lifestyle habits, physiological indicators, and diseases).

In 2021, our international collaborative study assessed data obtained from public biobanks regarding populations in Japan and the Netherlands [21]. This cross-sectional study included 28,265 spouse pairs from the Dutch Lifelines Cohort Study (recruited from 2006 to 2013) and 5,391 Japanese Tohoku Medical Megabank Organization (ToMMo) Cohort Study pairs (recruited from 2013 to 2016). Significant spousal similarities were noted in all the cardiometabolic risk factors (lifestyle habits, anthropometric traits, and diseases) investigated. For example, the odds ratios (ORs) [95% confidence interval (CI)] for spouse pairs were 4.60 (3.52–6.02) for current smoking, 2.83 (2.39–3.35) for current drinking, 2.76 (2.28–3.32) for sufficient physical activity, 1.20 (1.05–1.38) for hypertension, and 1.72 (1.47–2.02) for metabolic syndrome [21]. It is still possible that this result reflects the age similarity of spouses rather than environmental factors of spouses (e.g., cohabitation effect). To clarify the importance of mate cardiometabolic risk factors for similarity of environmental factors, it is necessary to examine whether they are observed in random male-female pairs while maintaining the age of the mate pair.

This study aimed to determine whether the similarities found between spousal pairs for cardiometabolic risks were also observed between random male-female pairs. Should the findings of this study support the hypothesis, targeted lifestyle-related interventions are likely to reduce cardiometabolic risk factors among spouses and prevent cardiometabolic diseases. Further, these findings could contribute to important future spousal studies on preventive strategies for cardiometabolic diseases. To investigate the study hypothesis, we analysed the data of more than 5,000 male-female pairs obtained from a large observational study in Japan [22, 23].

## Methods

### Participants

For this cross-sectional study, data were obtained from the Tohoku Medical Megabank (TMM) Community-based Cohort Study (hereafter referred to as TMM CommCohort Study) that was conducted in Miyagi Prefecture, northern Japan (this data was previously published elsewhere) [22, 23]. For the TMM CommCohort Study, participants were recruited for the baseline survey, using two approaches, between May 2013 and March 2016. Participants were recruited at the sites of the annual community health examinations conducted by local governments in Miyagi Prefecture for insured persons aged 40–74 years (Type 1 survey). Additionally, seven Community Support Centre facilities were established in Miyagi Prefecture for voluntary admission-type recruitment and for conducting participant health assessments (Type 2 survey). In the baseline survey, blood and urine samples were collected, as well as self-administered questionnaires that included information on lifestyle habits, medical histories, and family relationships. A series of physiological tests were also performed.

Individuals aged ≥ 20 years who resided in Miyagi Prefecture were eligible for participation in the study. For the TMM CommCohort Study, self-administered family relationship questionnaires were distributed and collected. All participants were required to answer the following question: “If you are living with family members who are participating in this TMM Project, please specify all their names and birthdays and your relationships with them (your spouse, father, mother, children, grandchildren, children’s spouses, father-in-law, mother-in-law, and others) with their consent.” Based on these responses, if a participant’s spouse was identified as a TMM CommCohort Study participant, then the spouse and the participant were defined as a spouse pair [21]. Using the spousal pairs, new male-female random pairs were generated by randomly placing women so that they would be the same age as the wife of the husband to ensure that the ages remained unchanged. In this way, we created virtual data of random male-female pairs based on the data of the original spouse pairs. After sorting women of the same age into groups, they were randomly shuffled using the SAS RANUNI function (SAS Institute, Cary, NC, USA) to create new male-female pairs that were the same age as the original spouse pairs. Owing to chance, a random male-female pair might have been also a spouse pair.

### Data collection and variables

Data on the following cardiometabolic risk factors were collected: anthropometric traits: height, weight, waist circumference and body mass index (BMI); systolic blood pressure (SBP) and diastolic blood pressure (DBP); glycated haemoglobin (HbA1c); lipid traits: total cholesterol (TC), triglycerides (TG), high-density lipoprotein-cholesterol (HDL-C) and low-density lipoprotein-cholesterol (LDL-C); and lifestyle factors. Cardiometabolic diseases such as hypertension, type 2 diabetes mellitus (T2DM), and metabolic syndrome were defined based on the collected data.

Specifically, well-trained staff measured the participants’ height, weight, and waist circumference. For waist circumference measurements, based on the diagnostic criteria for metabolic syndrome in Japan [24, 25], the assessment was conducted in the standing position, during light exhalation, and at the navel. If fat accumulation was marked and the umbilicus deviated downward, the assessment was made from the midpoint between the lower border of the ribs and the anterior superior iliac spine. BMI was calculated as weight (kg) divided by height (m) squared. BP was measured during municipal health checks (Type 1 survey) and/or at a Community Support Centre (Type 2 survey). For the Type 2 survey, BP was measured twice in the upper right arm using a digital automatic BP monitor (HEM-9000AI; Omron Healthcare Co., Ltd, Kyoto, Japan) after resting in a sitting position for at least 2 min. During the TMM CommCohort Study, non-fasting blood samples were collected using a standard protocol, and HbA1c levels were measured using latex agglutination turbidimetry. TC was measured with cholesterol dehydrogenase using an ultraviolet end (UV-End) method. HDL-C and TG were measured using direct and enzymatic methods, respectively. LDL-C was calculated using the Friedewald formula.

### Lifestyle factors

Lifestyle habits such as smoking, drinking, and physical activity levels were defined according to the self-reported questionnaires. To assess smoking status, the participants were categorized as current smokers, past smokers, or non-smokers. Drinking status was assessed by categorizing the participants as current drinkers or non-drinkers. Regarding physical activity, metabolic equivalent (MET) hours/day was calculated by multiplying the MET score for a specific activity by the number of hours spent on that activity per day. This study used the 80th percentile of the men’s MET hours/day as a cut-off for division based on physical activity levels into two categories, namely (1) sufficiently active (≥ 80th percentile of men’s MET hours/day) and (2) inactive (< 80th percentile of men’s MET hours/day) [21]. This cut-off value was applied to both men and women.

### Diseases

Hypertension was defined as SBP ≥ 140 mmHg or DBP ≥ 90 mmHg or the use of antihypertensive medication. Diabetes was defined as HbA1c ≥ 6.5% or the use of blood glucose-lowering medication. Participants were classified as having a metabolic syndrome if they met the first criterion and at least two of the following criteria [24, 25]: (1) waist circumference ≥ 85 cm in men and ≥ 90 cm in women, (2) hypertension (SBP ≥ 130 mmHg or DBP ≥ 85 mmHg or the use of antihypertensive medication), (3) hyperglycemia (HbA1c ≥ 6.0% or the use of blood glucose-lowering medication), and (4) high TG/HDL-C levels (TG ≥ 1.68 mmol/L [150 mg/dL] or HDL-C < 1.03 mmol/L [40 mg/dL] or the use of lipid-lowering medication).

### Educational level

Educational level was determined using the following seven categories: elementary school or junior high school; high school; vocational school; college or technical college; university; graduate school; or other. Educational level was then categorized as follows: low (elementary school or junior high school), medium (high school or vocational school), and high (college or technical college, university, and graduate school). The category “other” was treated as missing data.

### Statistical analyses

First, we tested whether there was a statistically significant difference in cardiometabolic risk factors (anthropometric traits, lifestyle habits, and diseases) between spouse pairs and random male-female pairs. For the statistical significance test, the categorical variable was a chi-square test. In the case of continuous variables, the Student-t test was performed (only triglycerides were tested by the Mann-Whitney U-test).

For continuous variables, Pearson’s correlation coefficient was used to determine correlations between random male-female pairs. To control for the potential confounding effect of age similarity, we also estimated the correlation coefficients for age-adjusted residuals among random male-female pairs. We used separate age-adjusted linear regression models for the men and women and saved the residuals after adjustment. The TG levels were log_10_-transformed for these analyses due to their skewed distribution [26].

For those using antihypertensive and/or lipid-lowering medication, the SBP, DBP, TC, and LDL values were adjusted to reconstruct the original population ranking of these individuals based on the expected treatment effects. For those using antihypertensive medication, 15 mmHg and 10 mmHg were added to the SBP and DBP values, respectively [21, 27]. For those receiving hyperlipidaemia treatment, the TC and LDL values were divided by 0.8 and 0.7, respectively [21, 28, 29]. Only for the analysis of HbA1c levels, the patients undergoing treatment for diabetes mellitus were excluded from the analysis as HbA1c levels are influenced by treatment. In this study, all cardiometabolic risk factors were excluded as outliers if they exceeded the mean ± 5 standard deviation.

For categorical variables, logistic regression analyses were performed to determine spousal concordance. To determine the risk in men, ORs and 95% CIs were calculated for current smoking, current drinking, sufficient physical activity, and the presence of diseases in their respective female partners, all of which were considered exposures. To adjust for age in the analyses, two new covariates were calculated: the average age of each spouse and the age difference between random male-female pairs. For random male-female pairs, ORs > 1.0 indicated higher degrees of concordance. All statistical analyses were performed using the software SAS, version 9.4 (SAS Institute Inc., Cary, NC, USA).

## Results

### Basic participant characteristics in the TMM CommCohort Study

Of the 76,955 individuals who were invited to participate in the TMM CommCohort Study, 54,952 agreed. After excluding those who withdrew consent, 5,391 spousal pairs were identified using the family relationship questionnaires. From these, 5,391 new random male-female pairs were identified.

Participant characteristics and results of the random male-female pairs in this study as well as the spouse pairs (reported in the Atherosclerosis journal 2021) are shown in Table 1. The mean ages of the men and women who participated in this study were 63.2 years and 60.4 years, respectively. Approximately two-thirds of the participants were ≥ 60 years old. More men indicated that they were current smokers, ever smokers, and current drinkers than women. The prevalence of hypertension, diabetes, and metabolic syndromes was higher among men than among women.

**Table1:** Characteristics of sociodemographic and cardiometabolic risk factors among spouse pairs and random male-female pairs

	Spouse pairs (Reported in our earlier study) [20]				Random male-female pairs (Exact age-match of spouse pairs)			
	Number of pairs	Husband	Wife	p-value	Number of pairs	Male	Female	p-value
**General characteristics**								
Mean age at baseline, years (SD)	5,391	63.2 (10.5)	60.4 (10.2)	< 0.001	5,391	63.2 (10.5)	60.4 (10.2)	< 0.001
Age group, years	5,391			< 0.001	5,391			< 0.001
20–39, n (%)		293 (5.4%)	346 (6.4%)			293 (5.4%)	346 (6.4%)	
40–59, n (%)		931 (17.3%)	1,373 (25.5%)			931 (17.3%)	1,373 (25.5%)	
60–69, n (%)		2,678 (49.7%)	2,995 (55.6%)			2,678 (49.7%)	2,995 (55.6%)	
≥70, n (%)		1,489 (27.6%)	677 (12.6%)			1,489 (27.6%)	677 (12.6%)	
Education level	5,254			< 0.001	5,245			< 0.001
Low, n (%)		643 (12.2%)	443 (8.4%)			643 (12.3%)	440 (8.4%)	
Medium, n (%)		3,141 (59.8%)	3,884 (73.9%)			3,140 (59.9%)	3,884 (74.1%)	
High, n (%)		1,470 (28.0%)	927 (17.6%)			1,462 (27.9%)	921 (17.6%)	
**Risk factors**								
Mean weight, kg (SD)	5,390	66.5 (9.6)	54.1 (8.7)	< 0.001	5,385	66.4 (9.5)	54.1 (8.7)	< 0.001
Mean height, cm (SD)	5,391	166.7 (6.0)	154.3 (5.6)	< 0.001	5,391	166.7 (6.0)	154.4 (5.6)	< 0.001
Mean waist circumference, cm (SD)	3,183	85.6 (8.2)	81.9 (9.2)	< 0.001	2,143	85.6 (8.1)	82.0 (9.0)	< 0.001
Mean body mass index, kg/m^2^ (SD)	5,198	23.9 (3.0)	22.7 (3.5)	< 0.001	5,009	23.9 (3.0)	22.7 (3.5)	< 0.001
Mean SBP, mmHg (SD)	5,106	129.7 (16.3)	125.9 (17.7)	< 0.001	4,840	129.7 (16.3)	125.9 (17.6)	< 0.001
Mean DBP, mmHg (SD)	5,106	78.9 (10.6)	75.2 (10.6)	< 0.001	4,841	78.9 (10.6)	75.2 (10.5)	< 0.001
Mean HbA1c, % (SD)	4,602	5.5 (0.5)	5.5 (0.4)	0.39	4,568	5.5 (0.4)	5.5 (0.3)	0.45
Mean total cholesterol, mg/dL (SD)	1,964	201.5 (33.5)	213.7 (35.9)	< 0.001	841	202.2 (33.6)	211.7 (36.2)	< 0.001
Median triglycerides, mg/dL [IQR, 25th, 75th]	5,384	104 [75–151]	88 [64–121]	< 0.001	5,307	107 [76–155]	91 [65–127]	< 0.001
Mean HDL-C, mg/dL (SD)	5,384	57.0 (14.5)	66.5 (15.5)	< 0.001	5,375	57.0 (14.4)	66.5 (15.5)	< 0.001
Mean LDL-C, mg/dL (SD)	3,191	118.9 (29.8)	128.1 (30.4)	< 0.001	2,143	125.8 (33.7)	136.9 (33.6)	< 0.001
**Lifestyle factors**								
Smoking status	5,313			< 0.001	5,313			< 0.001
Currently a smoker, n (%)		1,104 (20.8%)	248 (4.7%)			1,101 (20.7%)	248 (4.7%)	
Smoker in the past, n (%)		3,066 (57.7%)	670 (12.6%)			3,073 (57.8%)	671 (12.6%)	
Never smoked, n (%)		1,143 (21.5%)	4,395 (82.7%)			1,139 (21.4%)	4,394 (82.7%)	
Alcohol consumption status	5,356			< 0.001	5,354			< 0.001
Currently a drinker, n (%)		4,201 (78.4%)	2,187 (40.8%)			4,195 (78.4%)	2,186 (40.8%)	
Drinker in the past, n (%)		196 (3.7%)	71 (1.3%)			198 (3.7%)	70 (1.3%)	
Never a drinker, n (%)		959 (17.9%)	2,558 (47.8%)			961 (17.9%)	3,098 (57.9%)	
Sufficient physical activity (≥ 80th percentile of men’s MET hours/day)	5,342	1,074 (20.1%)	549 (10.3%)	< 0.001	5,318	1,063 (20.0%)	548 (10.3%)	< 0.001
Hypertension, n (%)	4,260	2,411 (56.6%)	1,770 (41.6%)	< 0.001	4,037	2,271 (56.3%)	1,719 (42.6%)	< 0.001
Type 2 diabetes, n (%)	3,469	504 (14.5%)	246 (7.1%)	< 0.001	3,355	501 (14.9%)	240 (7.2%)	< 0.001
Metabolic syndrome, n (%)	3,770	1,565 (41.5%)	1,026 (27.2%)	< 0.001	3,697	1,501 (40.6%)	1,014 (27.4%)	< 0.001

Abbreviations: DBP, diastolic blood pressure; HbA1c, glycated haemoglobin; HDL, high-density lipoprotein; IQR, interquartile range; LDL, low-density lipoprotein; MET, metabolic equivalent; SBP, systolic blood pressure; SD, standard deviation. For the statistical significance test, the categorical variable was a chi-square test. In the case of continuous variables, Student t-test was performed (only triglycerides were tested by the Mann-Whitney U test)

### Spousal similarities in cardiometabolic risk factors

Tables 2 and 3 show the correlations and concordances of cardiometabolic risk factors among random male-female pairs and spouse pairs. For the ages of men and women, the correlation coefficients were 0.934 for both the random male-female pairs and spouse pairs.

**Table 2:** Correlations of cardiometabolic risk factors among spouse pairs and random male-female pairs

Risk factors	Spouse pairs (Reported in our earlier study) ^[^20^]^		Random male-female pairs (Exact age-match of spouse pairs)	
	Simple correlation (95% CI)	Age-adjusted correlation (95% CI)	Simple correlation (95% CI)	Age-adjusted correlation (95% CI)
**Age at baseline**	0.934 (0.930, 0.937)	NA	0.934 (0.930, 0.937)	NA
** Weight**	0.119 (0.092, 0.145)	0.110 (0.084, 0.137)	0.008 (-0.018, 0.035)	-0.005 (-0.032, 0.002)
** Height**	0.297 (0.272, 0.321)	0.175 (0.149, 0.201)	0.161 (0.135, 0.187)	0.010 (-0.016, 0.037)
** Waist circumference**	0.132 (0.098, 0.166)	0.126 (0.092, 0.160)	0.005 (-0.037, 0.047)	0.005 (-0.038, 0.047)
**Body mass index**	0.134 (0.107, 0.161)	0.136 (0.109, 0.163)	-0.009 (-0.036, 0.019)	-0.007 (-0.035, 0.021)
**SBP**	0.163 (0.136, 0.190)	0.086 (0.059, 0.113)	0.075 (0.047, 0.103)	0.026 (-0.002, 0.054)
**DBP**	0.094 (0.067, 0.122)	0.073 (0.046, 0.100)	0.029 (0.001, 0.057)	0.036 (0.007, 0.064)
**HbA1c**	0.139 (0.110, 0.167)	0.080 (0.051, 0.109)	0.095 (0.066, 0.124)	0.021 (-0.008, 0.050)
**Total cholesterol**	0.074 (0.030, 0.118)	0.101 (0.057, 0.145)	0.035 (-0.033, 0.102)	0.071 (0.003, 0.138)
**Triglycerides**	0.109 (0.083, 0.136)	0.129 (0.102, 0.155)	-0.014 (0.041, 0.013)	0.002 (-0.025, 0.028)
**HDL-cholesterol**	0.098 (0.071, 0.124)	0.100 (0.073, 0.126)	0.002 (-0.025, 0.029)	0.003 (-0.024, 0.030)
**LDL-cholesterol**	0.084 (0.050, 0.119)	0.095 (0.060, 0.129)	0.021 (-0.022, 0.063)	0.032 (-0.011, 0.074)

Abbreviations: CI, confidence interval; DBP, diastolic blood pressure; HbA1c, glycated haemoglobin; HDL, high-density lipoprotein; LDL, low-density lipoprotein SBP, systolic blood pressure

**Table 3:** Concordance of cardiometabolic risk factors among spouse pairs and random male-female pairs

	Spouse pairs (Reported in our earlier study) [20]		Random male-female pairs (Exact age-match of spouse pairs)	
	Crude odds ratio (95% CI)	Age-adjusted odds ratio (95% CI)	Crude odds ratio (95% CI)	Age-adjusted odds ratio (95% CI)
**Smoking**				
Current smokers (vs. non-current smokers)	5.61 (4.33–7.30)^***^	4.60 (3.52–6.02)^***^	1.41 (1.05–1.88) ^*^	1.06 (0.78–1.43)
**Alcohol drinking**				
Current drinkers (vs. non-current drinkers)	2.76 (2.30–3.31)^***^	2.83 (2.39–3.35)^***^	1.04 (0.88–1.22)	0.94 (0.81–1.09)
**Sufficient physical activity** (vs. insufficient activity)	2.76 (2.28–3.32)^***^	2.76 (2.28–3.32)^***^	1.03 (0.83–1.29)	0.97 (0.77–1.21)
**Diseases**				
Hypertension (vs. absence)	1.75 (1.54–1.98)^***^	1.20 (1.05–1.38)^**^	1.61 (1.41–1.83) ^**^	1.07 (0.93–1.23)
Type 2 diabetes (vs. absence)	1.78 (1.29–2.42)^***^	1.34 (0.96–1.83)	1.50 (1.07–2.06) ^*^	1.08 (0.77–1.50)
Metabolic syndrome (vs. absence)	2.15 (1.85–2.50)^***^	1.72 (1.47–2.02)^***^	1.35 (1.17–1.57) ^**^	1.02 (0.88–1.20)

^*^P < 0.05, ^**^P < 0.01, ^***^P < 0.001

Abbreviations: CI, confidence interval. Non-current smokers include past smokers and those who never smoked

### Continuous risk factors

For anthropometric traits, the age-adjusted correlation coefficients ranged from − 0.007 (BMI) to 0.071 (TC) among random male-female pairs and from 0.073 (DBP) to 0.175 (height) among spouse pairs.

### Lifestyle factors

Regarding lifestyle habits, the crude OR (95% CI) for the logistic regression analysis was 1.41 (1.05 − 1.88) for current smokers, which demonstrated concordance between random male-female pairs. However, no significant association was noted after adjusting for age. Among spouse pairs, there was a strong spousal concordance for currently smoking (age-adjusted OR = 4.60). Further, there were higher degrees of spousal concordance for current drinking and sufficient physical activity (OR = 2.83, and OR = 2.76, respectively).

### Diseases

Regarding diseases, the crude ORs (95% CIs) in the logistic regression analyses were 1.61 (1.41 − 1.83) for hypertension, 1.50 (1.07 − 2.06) for T2DM, and 1.35 (1.17 − 1.57) for metabolic syndrome, suggestive of concordance between random male-female pairs. However, no significant associations were noted after adjusting for age. In contrast, there were strong spousal concordances for hypertension and metabolic syndrome among spouse pairs, with age-adjusted ORs (95% CIs) of 1.20 (1.05–1.38) and 1.72 (1.47 − 2.02), respectively. For diabetes, although the age-adjusted OR was as high as 1.34, no significant association was shown.

## Discussion

In our previous study, there were similarities in several cardiometabolic risk factors among spouse pairs [21]. Similarly, many previous studies have shown a high degree of statistically significant similarities or concordances among spouse pairs for cardiometabolic risk factors (anthropometric traits, lifestyle habits, and diseases) [7–15, 21]. The similarity of cardiometabolic risk factors in spouses might be the result of age similarity of the spouses. In order to clarify that the similarity of cardiometabolic risk factors in spouses was important for environmental factors (assortative mating and/or cohabitation effect), it is necessary to examine whether it is observed in random male-female pairs while maintaining the age of the spousal pairs. This may be owing to environmental factors playing a greater role in spousal similarities than genetic factors. Here, we hypothesized that, when using random male-female pairs rather than spouse pairs, the similarities in cardiometabolic risk factors will be reduced. Using random male-female pairs, we found few significant similarities in cardiometabolic risk factors, including continuous risk factors (anthropometric traits, blood indicators, blood pressure, HbA1c level, and lipid traits), lifestyle habits (smoking, drinking, and physical activity) and diseases (hypertension, T2DM and metabolic syndrome). These findings support our hypothesis that, when using random male-female rather than spouse pairs, the similarities in cardiometabolic risk factors were low. Therefore, similarities in cardiometabolic risk factors between spouse pairs observed in previous studies may be explained by environmental factors (assortative mating and/or cohabitation effect) rather than similarities by age similarity of the spouses.

Our previous study showed similarities in cardiometabolic risk factors between spousal pairs and showed age dependency in these similarities. Age-appropriate similarities between spouses were found in exercise habits (higher similarity in spousal pairs with increasing age) and smoking habits (higher similarity between young and old age groups) [21]. Since our results in this study for random male-female pairs did not show these similarities, possibly, these suggest enhanced similarities in cardiometabolic risk factors among spousal pairs.

Cardiometabolic risk factors in random male-female pairs showed significant similarities in current smoking status and prevalence of diseases (hypertension, diabetes, and metabolic syndromes) before adjusting for age. However, the association disappeared after adjustment for age. This may be due to the age-related relationship between the smoking rate, the prevalence of hypertension, the prevalence of diabetes, and the prevalence of metabolic syndromes in both men and women. Current smoking rates in men aged 20–39, 40–59, 60–69, and over 70 years are 40%, 31%, 20%, and 12%, respectively. For women aged 20–39, 40–59, 60–69, and over 70 years these rates are 10%, 10%, 3%, and 3%, respectively. The prevalence of hypertension was 12%, 42%, 60%, and 69% in men and 7%, 22%, 46%, and 59% in women of corresponding age groups. Prevalence of diabetes was 0%, 2%, 6%, and 9% in men and 0%, 1%, 5%, and 6% in women, correspondingly. Incidence of metabolic syndromes were 18%, 47%, 60%, and 63% in men and 2%, 11%, 34%, and 42% in women, respectively. Based on these figures, the trends for the prevalence rates in men and women by age grouping were consistent. Therefore, significant differences disappeared after adjustments for age in these parameters.

To the best of our knowledge, this is the first study to explore and compare spouse pairs and random male-female pairs. Furthermore, we used a large sample size of over 5,000 pairs to compare and determine concordance for various circulatory and metabolic indicators (blood indicators, lifestyle-related factors, and the prevalence of diseases). Spouse concordance may be explained primarily by assortative mating and cohabitation effects [16]. Random male and female pairs to which these two explanations did not contribute were of few similarities. On the contrary, the results revealed a strong association of cardiometabolic risk factors between spouse pairs by comparing the association of cardiometabolic risk factors between random male and female pairs.

Based on this knowledge, it was important to consider measures that contribute to the prevention and treatment of cardiometabolic diseases. After quantifying spousal concordance for cardiometabolic risk factors, it was suggested that prevention interventions targeting spouse pairs rather than individuals may be more effective [30]. For example, in a randomized controlled trial focusing on the weight loss effect of exercise training, both overweight spouses achieved significant weight loss [31]. Therefore, focusing on corrective intervention for lifestyle-related factors, which are correctable, may improve test values and even prevent diseases. Couples with unfavourable lifestyles may be able to correct their lifestyles and prevent illness by competing with and encouraging each other. Since most couples of a similar age have similar health statuses, it may be possible to prevent cardiometabolic-related diseases by actively encouraging one another to attend health checks (primary prevention) and disease screenings (secondary prevention) [32–34].

This study had some limitations. First, the male-female pairs in this study were selected from spouse pairs. An unmarried status has been associated with an increased frequency of unhealthy behaviour (especially in relation to smoking) and psychological issues (especially depression) [35, 36]. Participants in this study who were married likely had higher physical and psychological health levels compared with unmarried individuals. Regardless, in this study, the random male-female pairs were selected from a healthy population and had few significant similarities in cardiometabolic risk factors. We hypothesized that, if unmarried individuals were included, even fewer associations would exist. Second, participants who undergo health check-ups may have a higher-level health consciousness than those who do not [37], which could have caused a volunteer bias in our study. Third, for this study, we only targeted the general population in Japan. In our previous study, we performed an analysis using large-scale biobank data from two facilities, one in Japan and the other in the Netherlands. Spouse pairs showed similarities in several cardiometabolic risk factors at both facilities. As this study was conducted in a single country, the generalizability of the study findings may be limited. Moreover, it remains unclear whether the results of this study may be affected according to different races, which also limits its generalizability. Lastly, the results were after single randomisation, so these findings may just be due to chance.

## Conclusion

This cross-sectional study in Japan aimed to determine whether the similarities found between spousal pairs for cardiometabolic risks were also observed between random male-female pairs. There were few similarities in cardiometabolic risk factors among random male-female pairs. These findings support our hypothesis that, when using random male-female pairs rather than spouse pairs, fewer similarities in cardiometabolic risk factors were found. As spouse pairs may share environmental factors, intervention strategies that target lifestyle changes and lifestyle-related disease prevention may be effective among spouse pairs.

## Data Availability

The TMM data sharing policy is publicly available at http://www.megabank.tohoku.ac.jp/english/sample/. Request for use of the TMM biobank data for research purposes should be made by applying to the ToMMo headquarters. All requests are subject to approval by the Sample and Data Access Committee. Details are available upon request at dist@megabank.tohoku.ac.jp.

## Abbreviations

HbA1c: glycated haemoglobin
BMI: body mass index
BP: blood pressure
HDL-C: high-density lipoprotein-cholesterol
LDL-C: low-density lipoprotein-cholesterol
OR: odds ratio
CI: confidence interval
SBP: systolic blood pressure
DBP: diastolic blood pressure
TC: total cholesterol, TG triglycerides
T2DM: type 2 diabetes mellitus
